# Atrial Fibrillation after Inhalational Lung Injury: A Troubling Complication of a Rare Problem

**DOI:** 10.1155/2012/302057

**Published:** 2012-10-18

**Authors:** Ragesh Panikkath, Kenneth Nugent, Alejandro Perez-Verdia

**Affiliations:** Department of Internal Medicine, Texas Tech University Health Sciences Center, Lubbock, TX 79430, USA

## Abstract

Atrial fibrillation (AF) has been associated with lung diseases like pneumonia and chronic obstructive pulmonary disease but has only infrequently been associated with inhalational lung injury. We report two cases of resistant AF, which developed in young healthy manual laborers shortly after inhalational lung injury due to massive quantity of pesticides and anhydrous ammonia, respectively. They had no evidence of valvular or structural heart disease and did not have any previous medical problems. The AF was resistant to antiarrhythmic drugs and required pulmonary vein isolation in first patient and possibly the second patient who is currently being evaluated for this procedure. These arrhythmias may reflect direct myocardial injury during and after exposure. Alternatively, multiple mechanisms can cause atrial fibrillation in lung diseases, including hypoxemia, acidemia, inflammatory mediators, and structural changes in the atria and ventricle, and these could lead to AF in inhalational lung injury cases. AF needs to be excluded when patients present with palpitations after inhalational lung injury, especially since, if unrecognized, AF may lead to complications, like thromboembolic phenomenon and tachycardiomyopathy.

## 1. Introduction

Age adjusted multivariate analyses have shown that the major risk factors for atrial fibrillation (AF) are hypertension, heart failure, diabetes, and valvular heart diseases [1]. However, lung diseases, such as chronic obstructive pulmonary disease and pneumonia, have contributed to the development of AF in some patients. The inhalation of toxic chemicals can produce significant lung injury but only very infrequently causes AF. We now report information on two healthy young men with structurally normal hearts who presented with AF shortly after accidental inhalation of massive quantities of chemicals which resulted in lung injury.

## 2. Cases

**Figure 1:**
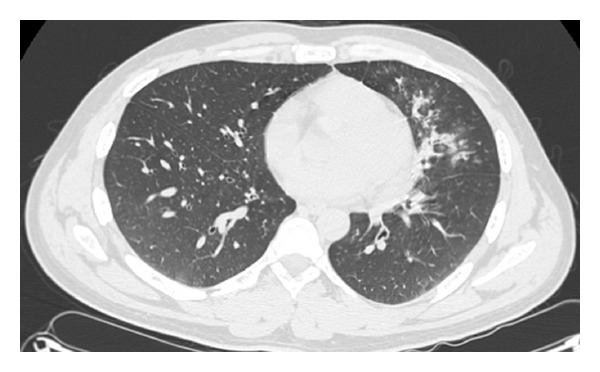
CT Chest of the first patient showing diffuse interstitial thickening, patchy infiltrates and areas of consolidation of the left lower lobe.

 The first patient is a 35-year-old man who worked as an insecticide sprayer on a potato farm. He was accidentally sprayed with over 900 gallons of a mixture of four agricultural chemicals when the valve holding the chemicals ruptured. The pesticides included a mixture of Venom insecticide (neonicotinoid), EPI-MEK meticide/insecticide (avernectin), Super-10 (permethrin), and Manzate Pro-Stick (fungicide). He presented with shortness of breath and hemoptysis to the emergency department immediately after the event. He subsequently developed diffuse interstitial lung disease, identified as desquamative interstitial pneumonia on lung biopsy (Figure 1). His pulmonary function tests (PFT) revealed a forced expiratory volume in first second (FEV1) 2.5 L (57% predicted), forced vital capacity (FVC) 3.3 L (61% predicted), and FEV1/FVC of 94% predicted consistent with a restrictive defect. On the next day after hospitalization, he started having recurrent episodes of symptomatic paroxysmal AF refractory to dronedarone. Echocardiogram revealed normal chamber sizes with a left atrial dimension of 2 cm and grade I-II diastolic dysfunction. A repeat study three months later at another facility reported a normal left ventricular systolic and diastolic function with an ejection fraction of 60–65%. He underwent bilateral antral pulmonary vein isolation due to an inadequate drug response. After ablation he is symptomatically better but remains oxygen dependent due to his lung disease. 

 The second patient is a 36-year-old man who was accidentally exposed to anhydrous ammonia when the tube transferring ammonia broke. He sustained lung injury and was brought to the emergency department. He developed persistent atrial fibrillation within hours after hospitalization. He was electrically cardioverted twice to sinus rhythm, but atrial fibrillation recurred in spite of treatment with propafenone and dronedarone. He recently underwent chemical cardioversion with dofetilide but had recurrence within one week of cardioversion. His echocardiogram revealed a left ventricular ejection fraction of 65–69%, normal diastolic function and chamber dimensions, and left atrial dimension of 2.7 cm. His thyroid stimulating hormone was within normal range. He is currently being evaluated for pulmonary vein isolation. Although this patient initially had respiratory failure after the exposure and required intubation, his lung function recovered. His PFTs later revealed a FVC of 6.2 L (110% predicted), FEV1 of 3.6 L (80% predicted), and a FEV1/FVC 72% of predicted. His initial X-ray revealed bilateral basilar infiltrates and atelectasis. His X-ray seven months later was within normal limits.

## 3. Discussion

 These patients were young healthy men without cardiac or respiratory disease prior to their inhalational lung injury. They did not have congenital or valvular heart disease, did not have hyperthyroidism, and did not use or abuse sympathomimetic drugs. The time course suggests that their AF developed as a consequence of inhalational lung injury, possibly secondary entry of high concentrations of chemicals and inflammatory mediators into the pulmonary veins with direct delivery into the left atrium. The atrial fibrillation in these patients was persistent and refractory to antiarrhythmic drugs. 

 Histological studies of atrial tissue in patients with AF demonstrate that these patients have myocarditis (64%), localized noninflammatory cardiomyopathy (17%), and patchy fibrosis (17%) [2]. In addition, fibrillatory activity causes ion channel and electrical remodeling which in turn lead to sustained reentry and permanent AF [3]. Chemical injury could initiate similar changes in the atria. Saadeh and coworkers reported that organophosphates or carbamates cause cardiac complications in up to 67% of subjects with poisoning [4]. The complications noted in this study included noncardiogenic pulmonary edema (43%), cardiac arrhythmias (24%), electrocardiographic abnormalities like prolonged corrected QT interval (67%), ST-T changes (41%) and conduction defects (9%), sinus tachycardia (35%), sinus bradycardia (28%) hypertension (22%), hypotension (17%), ventricular tachycardia (9%), and ventricular fibrillation. They reported that four patients had atrial fibrillation after exposure, but they also had hypoxia or ischemia which might have precipitated the atrial fibrillation. However, in another case report, where a person was exposed to methomyl dust (a carbamate pesticide), AF has been reported as a direct manifestation of the toxicity; the AF in this case was transient and reverted spontaneously to sinus rhythm within 24 hours [5]. The T wave changes associated with pesticide are possibly independent of the cholinesterase effect and directly related to the chemical (dose related) [6]. 

 High blood pressure, bradycardia, and cardiac arrest have been reported in humans as a result of acute exposure to highly concentrated aerosols of ammonium compounds [7]. George et al. report a case of severe ammonia poisoning, who suffered unexplained quadriparesis and severe sinus bradycardia [7]. The patient in this case report had a fatal cardiac arrest. AF has not been reported as a complication of anhydrous ammonia exposure to our knowledge. Myocardial fibrosis has been observed in a variety of mammals, like monkeys, dogs, rabbits, guinea pigs, and rats, after prolonged exposure to ammonia [8]. Since atrial fibrosis is one possible causes of persistent AF, it is possible that the refractory AF in our patient could be related to this mechanism. Ammonia is highly soluble and acts predominantly as an upper airway irritant [9]. Usually only minimal quantities reach the pulmonary alveoli, but higher concentrations of ammonia inhaled over a prolonged period of time can affect the lower respiratory tract causing edema, hemorrhage, ulceration, atelectasis, and respiratory failure [9]. Our patient had acute respiratory failure requiring intubation after his exposure but did not develop any long term pulmonary sequelae identifiable by PFTs. However, his AF continues to be a chronic problem.

 AF could also develop during acute lung disease as a consequence of acute sympathetic stress, hypoxemia, and inflammatory mediators. For example, seven patients (4.1% of 170) had new onset atrial fibrillation during an episode of pneumococcal pneumonia [10]. The authors of this study suggested that hypoxia caused the cardiac arrhythmias. However, at least one patient in this study had the hypoxia corrected by oxygen administration before AF occurred, and this suggests that other factors contributed to the development of arrhythmia. The mean age of these patients was 71 years. Both our patients were young without any co-morbidity. 

 AF has also been associated chronic lung disease. Shibata and coworkers studied 2917 subjects 40 years or older who participated in a community-based annual health survey. AF was associated with decreased FEV1 and FVC independent of age, gender, left ventricular hypertrophy, and levels of brain natriuretic peptide [11]. Shabaro et al. noted that patients with chronic respiratory insufficiency frequently had alterations in cardiac rhythm, including premature ventricular and/or supraventricular beats and less frequently AF, identified by Holter monitoring [12]. These studies suggest that the gas exchange and hemodynamic abnormalities associated with chronic lung disease cause or contribute to the development of arrhythmias. Our first patient developed acute and then chronic lung disease after pesticide exposure, and the mechanisms discussed below could contribute to the pathogenesis of his atrial fibrillation.

 Reduced pulmonary function could directly lead to the development of AF. Hypoxia, hypercarbia, respiratory and metabolic alkalosis, the release of inflammatory mediators, and the effects of drugs used to treat respiratory diseases could cause AF. Lung dysfunction causes hypoxia, and this increases sympathetic drive which could induce ectopy. In turn, ectopic foci in the pulmonary veins potentially trigger AF. The synergistic interactions among hypoxia, respiratory acidosis, and cor pulmonale have a proarrhythmic effect [12]. Metabolic disturbances like hypokalemia secondary to corticosteroids and diuretics can induce dispersion of refractory periods in myocardium and cause a proarrhythmia state. Theophylline and beta adrenergic agonists used for treating respiratory diseases also cause arrhythmias. High pulmonary artery pressures due to lung disease can produce dilatation of the right atrium and lead to perpetuation of atrial arrhythmias. Acute and chronic inflammation in some lung diseases releases inflammatory mediators in high concentrations into the pulmonary veins and then left sided cardiac chambers which potentially stimulate ectopic foci in the pulmonary veins. The inflammatory mediators implicated in lung disease, including C-reactive protein, fibrinogen, activated complement components, and cytokines (e.g., interleukin 6, interleukin 1, tumor necrosis factor-*α*) have also been associated with the development of AF [13]. 

## 4. Conclusion

 In summary, multiple mechanisms, especially if significant pulmonary impairment develops, could potentially contribute to the development of AF in patients with acute inhalational injury; these would include hypoxemia, acidemia, inflammatory mediators, and structural changes in the atria. The possibility of cardiac arrhythmias should be considered when these patients present to physicians. These arrhythmias potentially contribute to the physical limitations of these patients and have important complications, such as tachycardia mediated cardiomyopathy and thromboembolic phenomena.
